# Miscellaneous Notices

**Published:** 1843-06

**Authors:** 


### Miscellaneous Notices.

*Dr. Hayden's "Comments."*—As a matter of special courtesy and in compliance with a request made by Dr. H. H. Hayden to one of the editors of this Journal, and by him communicated to us, we consented to publish, without inspection, a paper from the former, purporting to be a review of our dissertation on the Diseases of the Maxillary Sinus. This request, we were informed, was made with a view of preventing us from exercising the editorial prerogative of noticing said "comments" in the same number of the Journal in which they made their appearance. Not wishing to avail ourself of any supposable advantage of "our good friend, the doctor," we consented to his request; and, had he confined his remarks to the dissertation in question, we should not, perhaps, have deemed any reply necessary, but inasmuch as he embraced the opportunity to censure, in no very measured terms, the manner in which this Journal has been conducted, a just regard for the reputation of those concerned in its editorship would seem to require that it should not be passed in silence.

In the first and second paragraphs of his "comments," the doctor says, "it was the duty of the editors, in publishing original essays, or reprints, to point out to the reader the correctness of the author's style, and the merits of the composition and subject, on the one hand, and on the other, to expose, with suitable comments, the errors of opinion, the evil tendencies of propagating or circulating mistaken views and unsound theories, either in science or morals, &c. &c." "We have often been at a loss to account for the apparent, manifest neglect, evinced in not attending to the important points so essential to the faithful and judicious fulfilment of the object of that Journal." He also charges "that several whole treatises, emanating from men of *high attainments and professional standing*, have been reprinted in the Journal, and, with the exception of one case, sent into the world for the *amusement* of the experienced, and the edification of tyros of the profession, with their errors unnoticed, their faults uncorrected, and their views and opinions handed over to be perpetuated through time, without the semblance of a review, or a single criticism from the legitimate organs of that work."

We leave to the learned author of the "comments" to reconcile the seeming inconsistency implied in the assertion that "men of high attainments and professional standing" are capable of writing for the "*amusement* of the experienced and the edification" only "of tyros." He certainly must be ignorant of both the editorial duties and position to suppose it possible for an editorial triumvirate to take upon itself the responsibility of becoming the censors of the profession, and the *ne plus ultra* of authority to decide on the justness of the various theories and doctrines advanced by surgeon dentists "*of high attainments and professional standing.*" Besides, how could these editorial reviews, decisions, and arbitrary opinions of the corps, give any more satisfaction to the learned author of the "comments," than the variant opinions of the authors themselves, especially when the editors should happen, as they might, to disagree with the doctor.

All scientific journals whose object is to elucidate and promote the cause of truth and sound science, must of necessity, be open to well written dissertations, essays and articles, allowing full liberty to the expression of opinion and freedom of discussion, if conducted within the limits prescribed by decorum. To institute a censorship, or exercise *ex cathedra,* a power of directing the readers to believe this and reject that part of any ably written article in the Journal, would indeed be an authority as monstrous as it would be unsatisfactory and nugatory in its proscriptive effects upon professional opinion. The supposition that a scientific journal can be advantageously and satisfactorily conducted on other than open and liberal principles has its origin in an ignorance of the duties of the editorial station.

We might, perhaps, more acutely feel the sweeping censures of Dr. Hayden, had not so many distinguished gentlemen of literary and scientific eminence, both in this country and Europe, spoken in terms of unmixed commendation of the manner in which the Journal is conducted, as well as of the matter which it generally contains. Such encouragements, coming from such sources, have constituted the highest rewards of the editorial duty of conducting the first Journal ever devoted wholly to the cause of dental surgery.

As it regards the doctor's criticisms on our dissertation, it is really amusing to follow him, lost and bewildered as he seems to be, in the plurality of "sinuses," and unlock their secret intent, by the aid of some of his key passages, which, like tell-tales, reveal his whole meaning. He modestly says; "we are bold to say, that *no one author, whose works have been published, has ever given* a full and accurate *physiological* view of these cavities, not even to the day, inclusive, on which the American Society of Dental Surgeons met in the principal lecture room of the medical college, in Mason street, Boston,"—and again, "Nay, we think that we are safe in saying, that our friend, the doctor himself, had, never during his long experience and *deep research,* been made acquainted with many of the peculiarities in the structure of these cavities, until the meeting of the society of dental surgeons in Boston, on the 20th of July, 1842. If to the contrary, however, it was acknowledged by a medical gentleman of high standing in Boston, previous to the meeting on the 20th of July, that he had never, before, been made acquainted with the peculiarities of these cavities, nor the cause of the frequent *recurrence* of disease in them, after being apparently healed, or relieved from every symptom of disease!"

The modesty of the learned and venerable writer of the "comments" seems to have been so great on this "punctum saliens," the core of his theme, that he not only leaves the idea, but his periods a little mystified or abridged. However, with all the precision that a Dervise would use in computing the precise moment, hour, day and year of the Hegira, we gain the stupendous fact that, up to the 20th day of July, 1842, "inclusive" of that day, the world, except in the lone manuscript in one trunk, were in utter ignorance of the structure and diseases of the maxillary sinus, and that thereafter, that is to say, on the 20th day of July aforesaid, "at 12 o'clock, M." science for the first time lighted up these caverns, and illuminated their before unknown regions. We like to be precise, and in speaking of the achievements of another person, have not, as Dr. Hayden evidently has when speaking of himself, any particular embarrassment from a sentiment of modesty.

Yet, after all, the doctor's memory will recall the expression of an eminent surgeon dentist on the occasion, that this light-giving essay contained doctrines to which he presumed few in the United States would be willing to subscribe.

Although the plan of treating affections of the maxillary sinus by injections through the nasal opening, as advocated by Dr. Hayden in his lecture, is inefficient and in most instances impracticable, as has been conclusively proven—we had hoped that the twenty years' "*deep* and *critical research*," of which he speaks, would have enabled him, in his "comments," to have thrown some new and valuable light upon the diseases of this cavity, but we regret to say that our expectations have not been realized. And although he has honoured our humble and unpretending dissertation with forty-eight pages of "comments," we fear he was actuated, in writing them, by other motives than a desire to point out the errors of opinion into which he seems to suppose we have fallen, or the promulgation of scientific truth.

But if Dr. Hayden has failed to adduce a single fact or argument capable of refuting any part of the doctrines of the dissertation in question, he has given us evidence that he is sportive and witty, as will be seen from the following phrases, extracted from his "comments:

He says, in true bird-hunting parlance, "my very esteemed friend, the doctor himself, has not even winged his snipe so as to bring him down;" again, he speaks of "the game," in connection with Sir Joseph Banks and the butterfly; and again, "as the game is started, it would betray less than the spirit of a huntsman to abandon the chase;" and yet again, he speaks of tumours less than "a bird shot." These sportsmanlike expressions are not to be overlooked in sientific writings, as they show not only the writer's favourite amusements, but also reveal the sources of his erudition. But we will pardon the doctor for having used them in his "comments," as they seem to be favourite expressions with him—he having employed most, if not all of them, if our memory serve correctly, in an article which he wrote many years ago. We hope, however, that his gun, like his pen, is not the identical weapon named by a witty author, (not Dr. Hayden's favourite Peter Pindar,)

> "The gun, that aimed at duck and plover,
> Shoots wide, and kicks its owner over."

We cannot adopt Dr. Hayden's plural interlineations in his extracts from our dissertation, as the use of the singular number, sinus, when treating of the subject generally, we consider more elegant and classic. We shall be under obligations to our "friend, the doctor," when next he may quote us, to do it correctly. In his quotation, on page 209, he writes, "this cavity (these cavities) *are* subject," &c. again, page 214, "abscesses," says our "friend, the doctor," is a different affection.

Dr. Hayden seems to regard it as a very "extraordinary coincidence" that we should both have read papers "treating of the same subject," at the last meeting of the American Society of Dental Surgeons, but to us is not at all extraordinary, inasmuch as he had been apprized, several weeks previously to the meeting, by a student of ours, that we were preparing, or intended to prepare, a dissertation on the diseases of the maxillary sinus, to read on the occasion. Dr. Hayden will also recollect that when called upon by a committee appointed by the Society to arrange the business of the meeting, on the first day of the session, for the subject of the paper which he proposed reading, he stated that he had brought several lectures with him, but had not then determined which he should deliver. The subject of our dissertation, in connection with those of several others, was immediately announced to the Society, and the next morning in three of the Boston papers. At 12 o'clock of the same day, Dr. Hayden read his lecture, and at 4 o'clock, we commenced the reading of our dissertation, and after having continued one hour, were, at our own request, excused from the further reading of it. A resolution was then offered, that so much of it as had been read, together with the remaining part, be published with the transactions of the Society. We mention this fact for the purpose of correcting the mis-statement made (unintentionally we presume) by Dr. Hayden, that it was ordered to be published only "in part." An amendment to this effect, we believe, was offered by Dr. H. but which not having prevailed, the resolution, as first presented was adopted. H.

### FOURTH ANNIVERSARY OF THE AMERICAN SOCIETY OF DENTAL SURGEONS.

THE occurrence of this event, on the third Tuesday of July next, in the city of Baltimore, should be kept in mind by all of the present members of the Society, and those who wish to become such. So great are the advantages of scientific association, from year to year, and so much strength and encouragement are gained by these annual re-unions of gentlemen engaged in the same laborious and useful profession, we have few fears that those who have been in attendance on the previous anniversary meetings of the society can, by any circumstance of ordinary moment, be induced to absent themselves from the one to be held in July next; yet some of the new members, and those who have never availed themselves of the privileges of their membership, may not think enough of the occasion to make those sacrifices which the ample benefits of the association will reward a hundred-fold.

Of the members of the American Society of Detal Surgeons, there cannot be one who has not, during the past year, learned something new in the profession, made some improvements, or encountered some anomalous cases in his practice, the knowledge of which would be an acquisition to the stores of dental information, and be a source of benefit to the Faculty at large. And the action of the Society indicates and secures a reciprocity of benefits; where a member brings to the common treasury the experience and improvements of only a single individual, he may carry away with him the gains and achievements in the art and science, effected by a hundred, besides all the social and scientific friendships and correspondences he may form, to be sources of information and delight to him through life.

The season of the year assigned to the anniversary meeting renders it peculiarly agreeable to southern members of the Society. It is precisely the time when Virginians, Carolinians, Georgians, Alabamians, Louisianians and Mississippians would choose to take an excursion to the middle and northern states; and to meet this brotherhood of the science, we feel confident they are ever ready to make sacrifices, which to all, north, south, west or east, the vacation from home professional duties may combine pleasure, business and instruction, in a degree that no other movement or organization could produce. The growing importance of the art, the security of regular, well-taught dentists against quackery and audacious empiricism requires this annual sacrifice, if such it may be called, at the hands of the associated members of this Society.

In view then of all these considerations, as well as that of the public good, we hope the attendance of the members of the association will be general; let it be a congress of science, with a full representation from every section of the country. H.

*Practice of Dental Surgery in Alabama.*—The letter from Dr. C. J. Clark, Secretary of the Jacksonville Alabama Medical Board, addressed to one of the editors of this Journal, and published in another part of the present number, affords pleasing evidence of an awakening solicitude to guard dental surgery from the encroachments so often made upon its domains by ignorant impudence and unprincipled quackery.

The law of the State of Alabama, quoted by our correspondent, is as ample as it can be, until a state association of dental surgeons shall have been established in that state—when the whole matter of guarding the profession and the public against imposition from unprincipled adventurers, claiming to be dentists, should be committed to it, as a responsible and chartered body, known in law, and having a deeper interest than any other portion of the community in allowing none but the educated and thoroughly qualified, to perform operations so connected as are the dental with health, beauty and organic freedom from pain and decay.

The insuperable objection to committing the interests of dental surgery, into the keeping of the general surgeon and the physician, is, that gentlemen who are only medically educated, as far as it regards dental surgery, are oftentimes as ignorant as the most unlearned. The medical colleges have never taught this branch of surgery, in its most important and difficult operations; and hundreds of students are graduated yearly who do not really know how to extract a tooth scientifically. The addition of a regularly educated and authorised dentist to the committee of examination, when such a dentist could be found, tends to give more security to the public; still the security would be far greater if the whole duty and responsibility rested where it should, in a state association of dental surgeons.

Yet we would not be understood as underrating, or implying any thing to the disparagement of the scientific and talented members of the medical board in Alabama, who, as yet, have power, by law, to guard themselves and the community from empiricism. Much may be done, even in this way; but the true remedy lies in the general union of educated dentists in a central association, aided and sustained by state societies. Such, acting with as much power from the state laws as surgeons and physicians have, will be able to make the profession honorable, respectable and useful. H.

*Omission.*—In the account which we gave of the proceedings of the last meeting of the American Society of Dental Surgeons, in the first number of the present volume of the Journal, we inadvertently neglected to state that Dr. E. Baker, of New York, declined being a candidate for re-election to the office of treasurer. We should have made this correction in the succeeding number of the Journal, but the notice which we had prepared of it having been mislaid, the circumstance escaped our attention until after the publication of the third number, and believing the statement of the fact to be due to Dr. Baker, who served the society so faithfully in that capacity for two years, we mention it now.

*Another Tooth injected with red blood.*—Among some dental pathological specimens recently sent to us by Dr. E. Taylor, surgeon dentist, of Natchez, Miss. is one, a part of which is beautifully injected with red blood, presenting a precisely similar appearance to the tooth exhibited to us a few months since, by Dr. E. Maynard, of Washington City, and noticed in a former number of the Journal. Accompanying the morbid specimens which Dr. T. had the kindness to send to us, were several fossil teeth, taken from a mound near Natchez, and on the side of one of these, partly between the bifurcation of its roots, and nearly an eighth of an inch from the termination of the enamel of the crown of the tooth, is an enamelled protuberance, the size of a large pin's head. H.

